# Apparent Ploidy Effects on Silencing Are Post-Transcriptional at HML and Telomeres in *Saccharomyces cerevisiae*

**DOI:** 10.1371/journal.pone.0039044

**Published:** 2012-07-09

**Authors:** Jenny M. McLaughlan, Gianni Liti, Sarah Sharp, Agnieszka Maslowska, Edward J. Louis

**Affiliations:** Centre for Genetics and Genomics, Queen’s Medical Centre, University of Nottingham, Nottingham, United Kingdom; Tulane University Health Sciences Center, United States of America

## Abstract

The repression of genes in regions of heterochromatin is known as transcriptional silencing. It occurs in a wide range of organisms and can have importance in adaptation to the environment, developmental changes and disease. The model organism *Saccharomyces cerevisiae* has been used for many years to study transcriptional silencing, but until recently no study has been made in relation to ploidy. The aim of this work was to compare transcriptional silencing in haploids and diploids at both telomeres and the hidden mating-type (*HM*) loci. Transcriptional silencing was assayed, by growth on 5-fluoroorotic acid (5-FOA) media or by flow cytometry, on strains where a telomere or *HM* locus was marked. RNA levels were measured by quantitative RT-PCR to confirm that effects were transcriptional. 5-FOA assays and flow cytometry were consistent with transcriptional silencing at telomeres and at *HML* being reduced as ploidy increases which agreed with conclusions in previous publications. However, QRT-PCR revealed that transcriptional silencing was unaffected by ploidy and thus protein levels were increasing independently of RNA levels. At telomere XI left (XI-L), changes in protein level were strongly influenced by mating-type, whereas at *HML* mating-type had much less influence. The post-transcriptional effects seen in this study, illustrate the often ignored need to measure RNA levels when assaying transcriptional silencing in *Saccharomyces cerevisiae.*

## Introduction

In *Saccharomyces cerevisiae* transcriptional silencing occurs at three regions of the genome: some telomeres, the hidden mating-type left (*HML*) and hidden mating-type right (*HMR*) loci, and the rDNA locus [1]. The mechanism of silencing at *HM* loci and telomeres is similar. The process is initiated by the binding of proteins to specific sites on the DNA which leads to recruitment of the silent information regulator proteins (Sir2-4); it is thought that Sir2 then deacetylates histones, allowing the spread of Sir protein binding and the formation of a heterochromatin-like structure [2], [3]. If any of Sir2-4 genes are deleted, telomeric and *HM* silencing are lost [1]. The other Sir protein, Sir1, is not essential for silencing, but its deletion results in a mixed population of silenced and non-silenced *HM* loci [4].

At chromosome ends, Rap1 binds to telomeric repeats, and there are sites for the origin recognition complex (ORC) and Abf1p in the subtelomeric core X region. Telomere-associated silencing is also known as telomere position effect (TPE) [5]. It is observed at some, but not all, chromosome ends and is maximal at the core X region [6], [7]. It is as yet unknown why yeast chromosome ends behave differently in terms of silencing and it is not known what significance TPE has to this organism.

The chromatin structure of *S. cerevisiae* chromosome ends has been analysed by micrococcal nuclease digestion of strains marked with a *URA3-yEGFP* reporter adjacent to core X. This revealed distinct differences between silenced and non-silenced ends: there was heterochromatin-like structure on the centromeric side of the reporter only at the silenced end and the *URA3* promoter was also more closed at this end [6].

The *HM* silencing is initiated by the binding of proteins to the flanking E and I silencer elements. These contain binding sites for ORC, Rap1p, and Abf1p [1]. The *HM* loci contain silenced copies of the yeast mating-type genes. Expressed copies of these genes occur at the mating-type locus *MAT*; a1 and a2 are expressed from *MATa* and alpha1 and alpha2 from *MATalpha*. Haploid yeast have either *MATa* or *MATalpha* and mating occurs between opposite types, thus diploid cells have both *MATa* and *MATalpha* and have a non-mating phenotype. The function of the *HM* loci is to allow haploid yeast to undergo a switch of mating-type so that mating can then occur between haploid cells that were originally of the same type. In order to switch mating-type, *MAT* is cut by an endonuclease encoded by the *HO* gene and then the opposite mating-type is copied from the appropriate *HM* locus [8].

Proteins expressed from the *MAT* locus/loci serve to activate or repress limited sets of genes, either directly or indirectly, resulting in some important mating-type defined differences between cells [9]. In cells which are heterozygous for *MAT*, the a1/alpha2 repressor is formed; this complex represses a set of haploid specific genes. If haploid cells did not maintain silencing of the *HM* loci, the cells would express both ***a*** and *alpha* genes and be unable to mate. This occurs for example if the *SIR3* gene is deleted from haploid cells. The silenced chromatin structure at the *HM* loci also prevents these sites from being cut by the *HO* endonuclease [8], [10], [11]. Diploid cells with heterozygous mating-type do not express the *HO* endonuclease since it is one of the genes repressed by the a1/alpha2 repressor.

In *S. cerevisiae* transcriptional silencing is typically measured by placing a reporter such as *URA3* at the relevant position and then monitoring the growth on selective plates. For example, strains with the *URA3* reporter are grown on 5-FOA plates which only permit growth if this gene is silenced [12]. We also assayed silencing by flow cytometry, measuring URA3-yEGFP expressed from telomere XI-L or YFP expressed from *hml*::*YFP*. Previous work has used flow cytometry to study transcriptional silencing, with increased fluorescence taken to show a decrease in silencing [13]. Flow cytometry has advantages over the plate assay in that it measures the level of expression in every cell.

Silencing in yeast has generally been studied in haploid cells. We were interested to make a comparison of the levels of silencing in haploids and diploids both at telomeres and at the *HM* loci. There were two reasons for undertaking this study: firstly, wild-type yeast (*HO*) naturally exist for most of the time in the diploid state, and secondly, when yeast is being used as a model organism the purpose is usually to achieve greater understanding of biology of diploid organisms such as man. Thus it is of importance to understand the status of transcriptional silencing in diploid yeast. Using 5-FOA assays, flow cytometry and quantitative RT-PCR we measured telomeric and *HML* silencing in haploids and higher ploidies, leading to some unexpected findings.

## Results

### Levels of a Telomeric Reporter Protein are Increased in Diploids

In haploid yeast cells, some chromosome ends are silenced while others are not [6], [7]. In order to compare telomeric silencing in haploids and diploids, the *URA3-yEGFP* reporter was positioned at the left end of chromosome XI (a silenced end) in a haploid strain and this strain was used to create a diploid with a single copy of the chromosome labeled. Silencing was measured using the 5-FOA assay. Growth on 5-FOA plates was less in diploids than in haploids, suggesting that telomeric silencing is reduced in diploids (Table 1).

**Table 1:** Percentage *URA3* repression at telomeres as measured by 5-FOA assay.

Species	Strain*	Telomere	Ploidy and mating-type			
			n a or α	2n a/α	2n a or α	n a/α
*S. c.*	S288C	XI L	40	7	17	–
*S. c.*	S288C	VII L	40	5.6	28	–
*S. c.*	Y55	VII L	52	11	–	0
*S. p.*	YPS138	VII L	60	1.5	–	–

* Strain or strain background. *S. c.*: *Saccharomyces cerevisiae*, *S. p.*: *Saccharomyces paradoxus*. XI-L: left end of chromosome XI.

**Figure 1:**
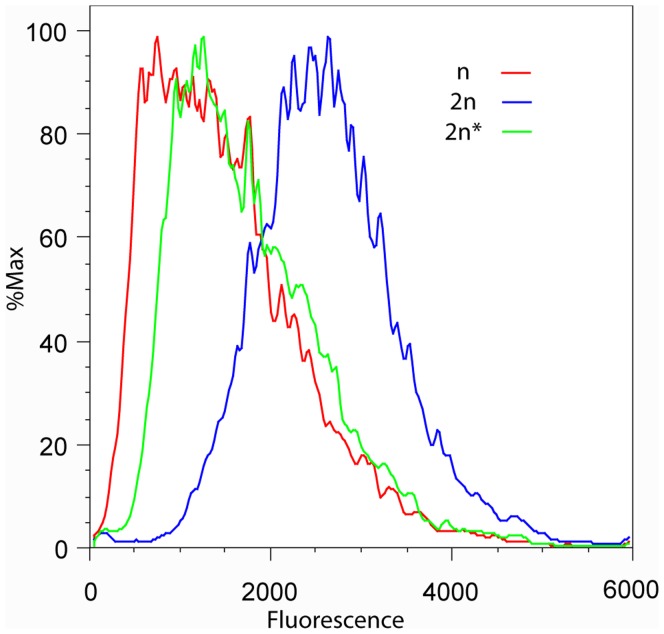
Level of telomeric reporter protein increases in diploids in a mating-type dependent manner. Yeast strains containing a single *URA3-yEGFP* reporter adjacent to core X at the left end of chromosome XI had fluorescence levels measured by flow cytometry. Histogram plots are for three strains: haploid (n, red), diploid (2n, blue), diploid with *MATa* deleted (2n*, green).

The fluorescent protein tag in the reporter construct allowed expression levels to be compared by flow cytometry as well. The resulting fluorescence intensity histograms clearly showed that the average fluorescence was greater in the diploid than the haploid cells (Figure 1). Thus the flow cytometry and 5-FOA assay results were both consistent with silencing at telomeres being reduced in diploid compared to haploid cells.

5-FOA assays were also carried out on strains in which *URA3* was positioned at the truncated VII-L telomere; this is another chromosome end which is known to be silenced in haploids [5]. Measurements were performed in two different strain backgrounds of *S. cerevisiae* and in *S. paradoxus* [14]. The results were again consistent with there being less silencing in diploids than in haploids and this reduction was variable between different genetic backgrounds (Table 1).

### Levels of a Telomeric Reporter Protein are Strongly Influenced by Cell Mating-type

The majority of differences between haploid and diploid yeast are determined by their mating-type [9], hence it was thought likely that this would also be responsible for the apparent differences in telomeric silencing between haploids and diploids. To investigate this possibility one copy of *MAT* was deleted from the diploid strain to give it a haploid mating-type. Flow cytometry and 5-FOA assays of the new diploid strain showed that it had increased 5-FOA resistance and decreased URA3-yEGFP fluorescence. The reporter levels were now more similar to those of the haploid cell (Figure 1).

Mating-type dependent changes in 5-FOA resistance were also observed in the strains with the *URA3* reporter positioned at VII-L (Table 1). In the S288C diploid, deletion of a single copy of *MAT* increased 5-FOA resistance and in the Y55 haploid, heterozygosity for *MAT* resulted in no detectable growth on 5-FOA. Thus, influence of mating-type on levels of telomeric reporter protein is not unique to one particular telomere or to one strain background.

### Changes in Levels of Telomeric Reporter Protein are Consistent with Chromatin Changes Found at its Promoter

**Figure 2:**
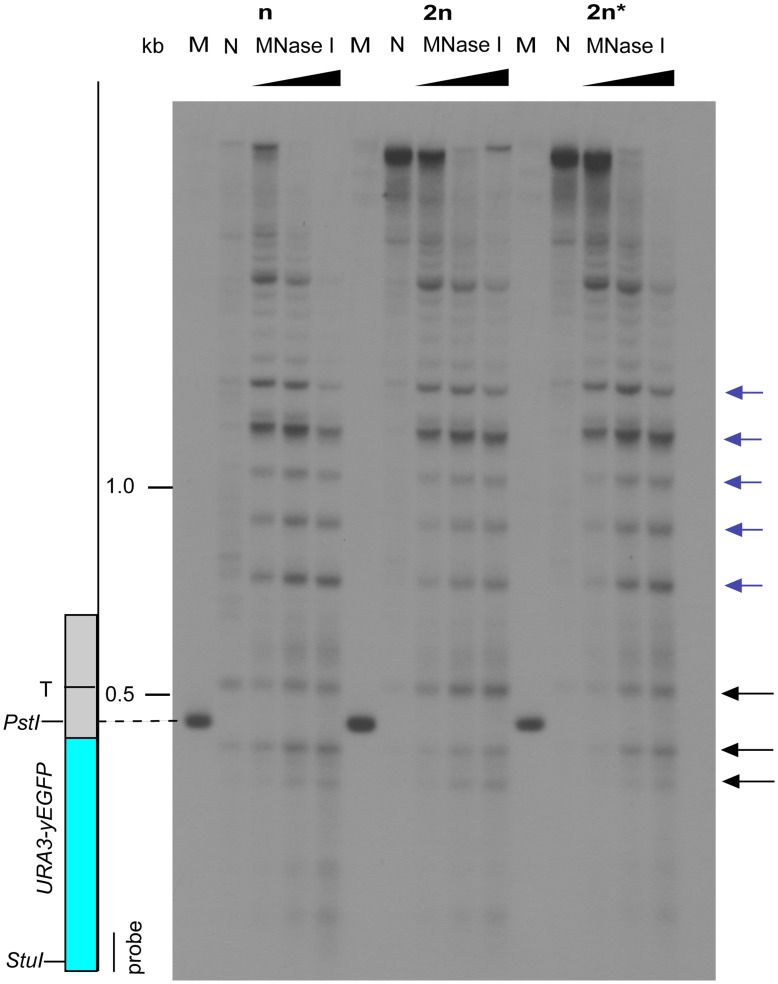
Mating-type dependent changes at the promoter of the *URA3-yEGFP* telomeric reporter. Southern blots of micrococcal nuclease I digests of chromatin from haploid (n), diploid (2n) and diploid *mata*-Δ (2n*) cells with *URA3-yEGFP* reporter at telomere XI-L. Position of *URA3-yEGFP* reporter is shown: promoter as grey box with TATA box (T) and ORF as blue box. Centromeric to the reporter are the regularly spaced hypersensitive sites typical of heterochromatin (blue arrows) and around the promoter are three hypersensitive sites whose relative intensity varies (black arrows). Control MNase I digests of deproteinized DNA (N), marker bands generated by digestion with *Stu*I and *Pst*I (M), and sizes on blot (kb) are also shown.

**Figure 3:**
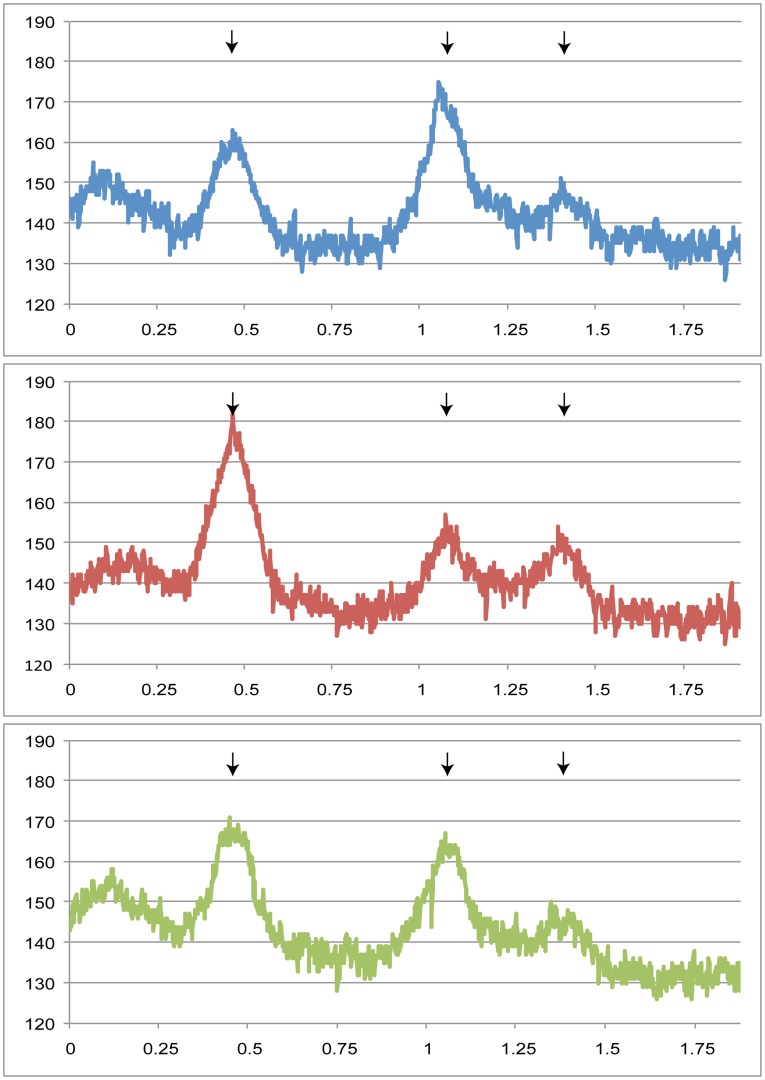
Measurement of promoter band intensities on chromatin blot confirmed mating-type dependent changes. Measurements were made on the autoradiograph of the chromatin blot of strains with a telomeric reporter at XI-L. Intensities were measured along a vertical line drawn through the three promoter bands produced by digestion with the highest concentration of MNase I. The graphs show intensity versus distance in inches along the line. Arrows indicate the band positions. The left hand peak corresponds to the top promoter band on the auroradiograph. Strains on graphs: haploid (blue), dipoid (red), diploid *mata-*delta (green).

Previous work, using micrococcal nuclease digestion to analyse chromatin structure, showed changes at the *URA3* promoter when the *URA3-yEGFP* reporter was positioned at a silenced end (XI-L), compared to the same reporter being positioned at the *URA3* native locus or at a non-silenced end (III-R) [6]. The relative intensity of three promoter associated bands was found to reflect the state of the promoter: the central band was most intense when the promoter was closed (XI-L) and it was less intense when the promoter was open (III-R and at the native *URA3* locus). Also, the nature of the chromatin on the centromeric side of the reporter correlated with the silencing state in that phased nucleosomes were only seen at XI-L [6].We used the same approach to compare chromatin structure around the reporter at XI-L in haploid, diploid and diploid cells with one copy of *MAT* deleted (Figure 2); the haploid strain was the same as that used by Loney *et al* [6]. It was found that the phased nucleosomes on the centromeric side of the reporter were maintained in all three cases (blue arrows Figure 2) but the chromatin at the promoter exhibited slight but reproducible changes (black arrows Figure 2). The haploid cells had the band intensity pattern resembling that of a closed promoter with the central band being most intense, whereas the diploid cells resembled an open promoter with the first band being most intense, and the diploid *mata*-delta cells had a promoter state more similar to that of haploid cells (Figure 2). The intensities of the promoter bands were quantified to confirm this observation. (Figure 3).

### Protein Levels of a Reporter at *HML* Increase with Ploidy, but are Almost Unaffected by Mating-type

**Figure 4:**
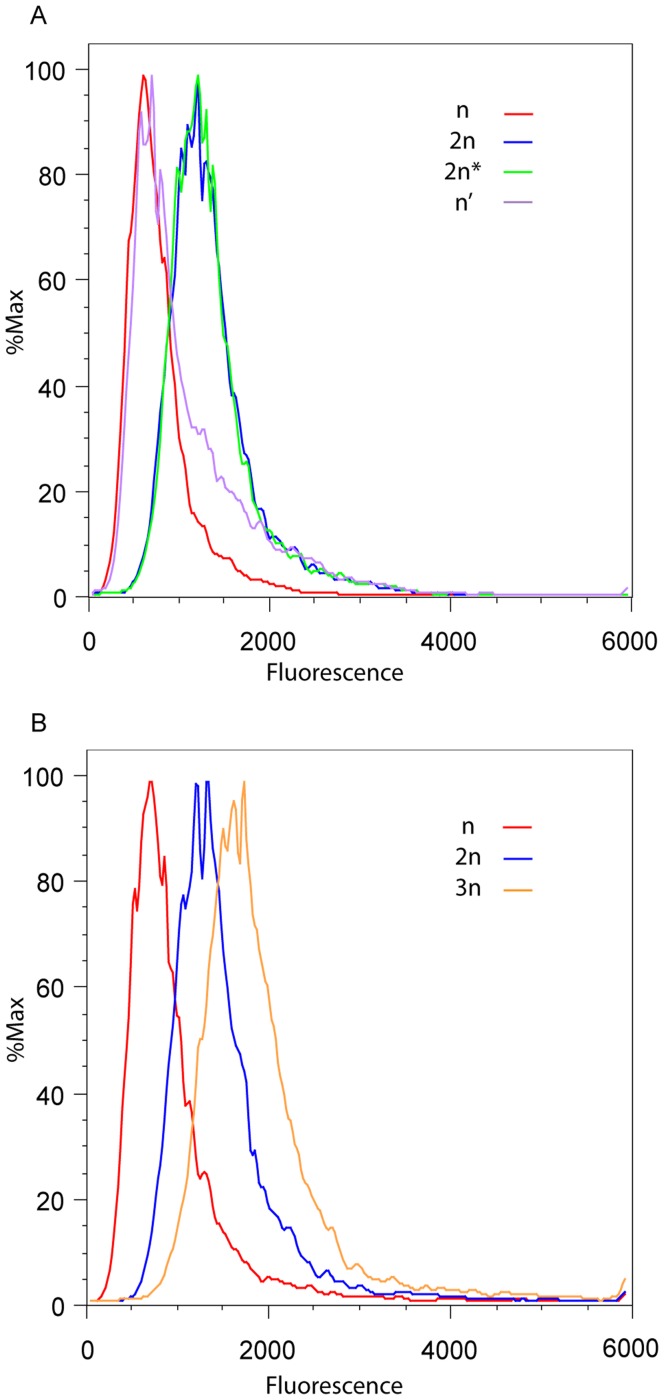
Level of protein from *hml::YFP* depends on ploidy but not mating-type. Yeast strains containing a single *YFP* reporter at *hml* had fluorescence levels measured by flow cytometry. (A) Strains on histogram plot: haploid (n, red), diploid (2n, blue), diploid with one *MAT* deleted (2n*, green) and haploid made heterozygous for *MAT* (n’, purple). (B) Strains on histogram plot: haploid (n, red), diploid (2n, blue), triploid (3n, orange).

The other regions of yeast chromatin which maintain transcriptional silencing by a similar mechanism to telomeres are *HML* and *HMR*, thus it was of interest to determine the response of an *HM* reporter to changes in ploidy and mating-type. Expression at *HML* was measured by flow cytometry using strains previously used by Xu *et al* [13] and derived strains. The strains had most of the *alpha2* ORF and all of the *alpha1* ORF at *HML* replaced by the reporter which consisted of the ORF for yellow fluorescent protein (*YFP*) flanked by the *URA3* promoter and terminator. Diploid cells were created in which only one copy of *HML* contained the YFP reporter. Comparison of haploids and diploids by flow cytometry revealed higher fluorescence in the diploid cells (Figure 4A). To examine the effects of mating-type on reporter protein levels, changes were made to the *MAT* status of haploid and diploid cells: *MATa* was integrated into the haploid cells to make them heterozygous for *MAT*, and one copy of *MAT* was deleted from diploid cells to give them a haploid mating-type. These alterations of mating-type had little effect on the amount of reporter protein per cell, although when haploid cells were made heterozygous for MAT there was a small increase in the number of cells with higher levels of protein (Figure 4A). These results suggest that levels of the reporter protein from *HML* were being altered by a mainly ploidy-controlled factor rather than by mating-type.

To further confirm that ploidy was influencing the levels of reporter, triploid strains were created by mating the *mata*Δ::*kanMX* diploid strain with a *MATa* haploid which was wildtype for *HML*. Flow cytometry showed higher fluorescence for triploids than for diploids (Figure 4B).

### Increase in Fluorescence From *hml::YFP* with Ploidy is Not Due to Insufficiency of Essential Silencing Proteins Sir3 or Sir4

**Figure 5:**
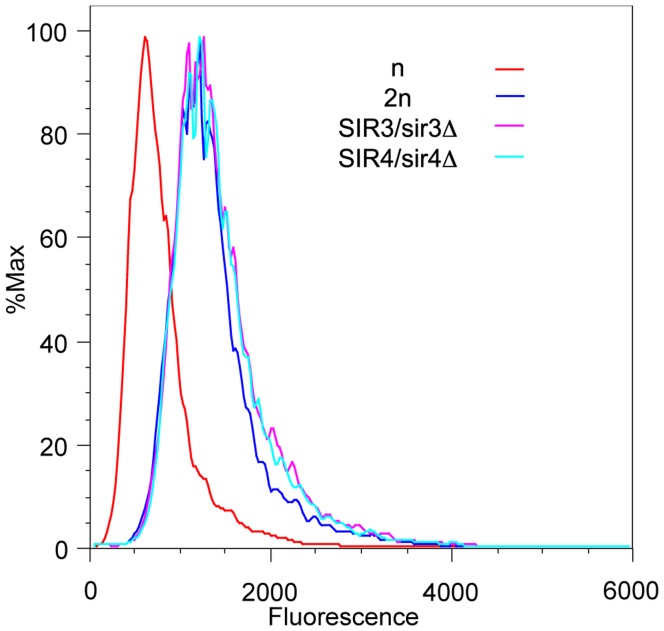
Neither Sir3 nor Sir4 are limiting at *HML* in diploid cells. A single copy of *SIR3* or *SIR4* was deleted from diploid cells and fluorescence levels were measured by flow cytometry. Strains on histogram plot: haploid (n, red), diploid (2n, blue), *SIR3*/*sir3-*Δ (pink), *SIR4*/*sir4-*Δ (turquoise).

Cell volume increases with ploidy [15], so it was thought that the levels of an essential silencing protein might become insufficient as ploidy increased. It has been found previously that silencing at the *HM* loci can be affected by the dosage of Sir proteins [16], [17]. Sussel *et al* found that presence of two copies of *SIR4* in a haploid increased silencing at *HMR*, or at *HMR* with the autonomously replicating sequence consensus sequence (ACS) deleted from the silencer (*hmr*Δ*A*), and they also found that deletion of 1 copy of *SIR4* from a diploid decreased silencing at *hmr*Δ*A* [17]. To investigate whether insufficiency of Sir protein might be the cause of the apparent decrease of silencing with ploidy, a single copy of *SIR3* or *SIR4* was deleted from diploid cells. It was found that this did not change the levels of YFP, suggesting that neither Sir3p nor Sir4p are limiting in diploids (Figure 5).

### Levels of Fluorescence From *hml::YFP* are Correlated with the Size of the Cell

**Figure 6:**
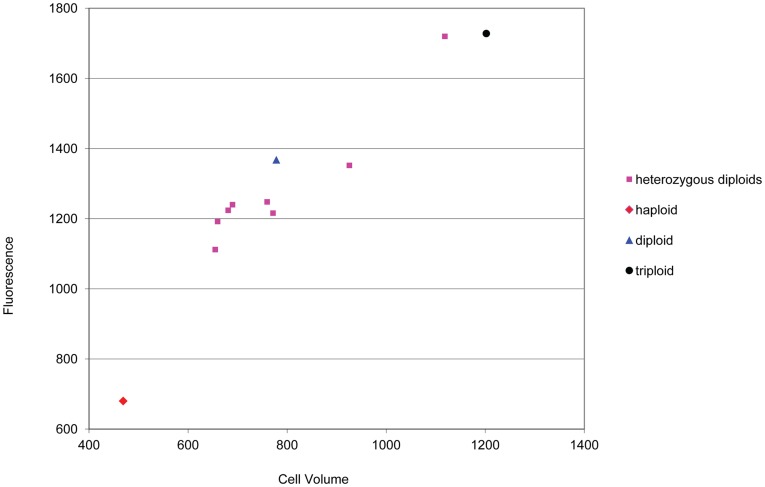
Levels of protein from *hml::YFP* reporter increase with cell size. Diploid strains with a range of cell size were created by mating appropriate strains from the haploid Yeast Deletion Collection [19] to the haploid strain containing the *hml::YFP* reporter. YFP levels were measured by flow cytometry in these strains (pink squares) and in haploid (red diamond), diploid (blue triangle) and triploid (black circle) strains. Mode fluorescence was plotted versus cell volume in µm^3^ (measured by microscopy).

The effects of ploidy on fluorescence from *hml::YFP* may still be due to the increase in cell volume. To test this theory we created several diploid strains which were heterozygous for genes known to have a dosage effect on cell size [18]. These strains were derived by mating chosen strains from the haploid Yeast Deletion Collection [19] to the haploid strain containing the *HML* reporter (*hml::YFP*). YFP levels were measured by flow cytometry and cell volume was calculated using microscope images of the same samples. The results showed a clear linear relationship between YFP levels and cell volume (Figure 6).

### Increase of HML Reporter Protein with Ploidy is not Due to a Decrease in Transcriptional Silencing

**Figure 7:**
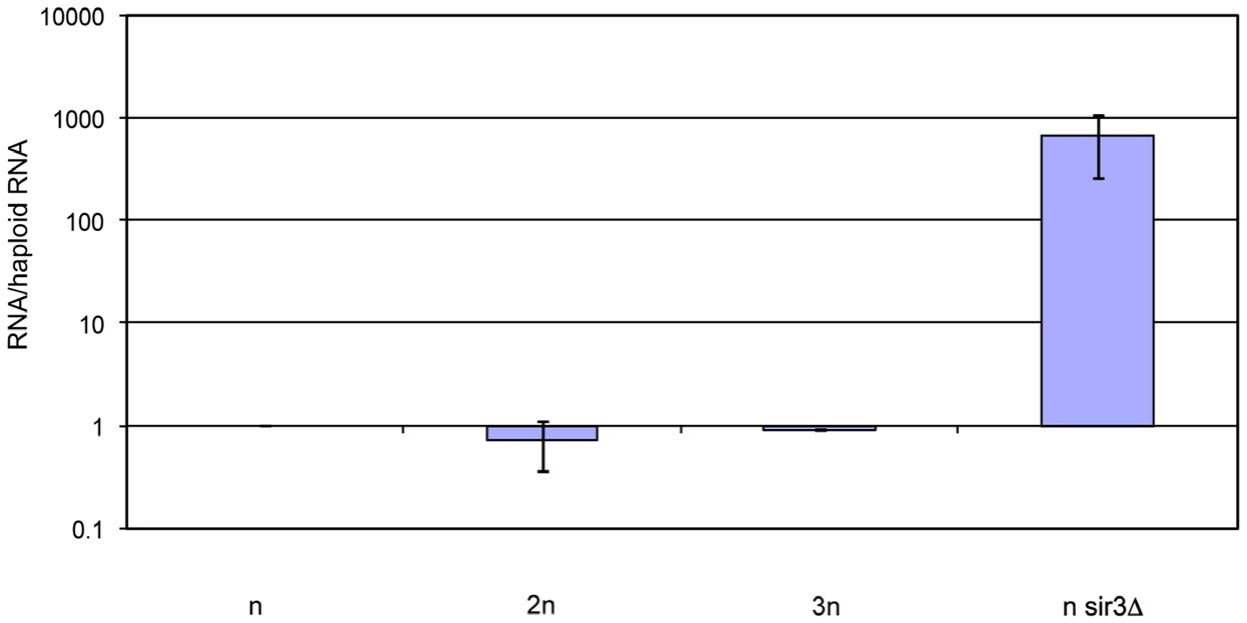
Transcriptional silencing at *HML* does not change with ploidy. mRNA levels were measured by QRT-PCR and compared to the levels in haploid cells. The values plotted are ratios to the amounts in the haploid cells. They are mean values from 3 separate RNA extractions. The error bars show 2 standard deviations.

The assays used so far to measure silencing had only given an indirect measure of transcriptional silencing in that they provided a measure of protein levels. Clearly protein levels can be influenced by many factors apart from transcription levels. In order to measure transcription more directly, RNA was extracted from equal numbers of haploid, diploid, triploid and haploid *sir3-*Δ cells and the relative amounts of YFP RNA were determined by quantitative RT-PCR. The results showed that amounts of YFP RNA per cell did not change with ploidy. Cells with *SIR3* deleted are known to have no silencing [1] and the haploid *sir3-*Δ cells did indeed have much higher levels of YFP RNA than the other cells (Figure 7).

### Transcriptional Silencing at Telomeres is Unaffected by Ploidy

**Figure 8:**
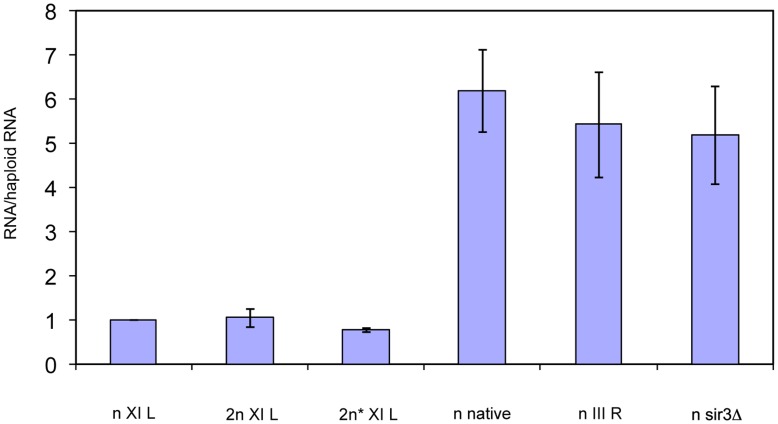
Transcriptional silencing at telomeres does not change with ploidy. mRNA levels were measured by QRT-PCR and compared to the levels in haploid cells. The values plotted are ratios to the amounts in the haploid cells. They are mean values from 3 separate RT-PCR reactions. The error bars show 2 standard deviations. The strains had the reporter positioned at XI-L (n XI L, 2n XI L, 2n* XI L, n *sir3*Δ), at *URA3* native locus (n native), or at III-R (n III R).

QRT-PCR for yEGFP was performed on strains labeled with *URA3-yEGFP* at telomere XI-L. As controls, the levels of yEGFP RNA were also measured in the haploid cells with *SIR3* deleted (no transcriptional silencing) and in strains where the *URA3-yEGFP* was at the *URA3* native locus or at a non-silenced end (III-R). The RNA levels from the XI-L reporter were the same in haploid cells, diploid cells and diploid cells with one copy of *MAT* deleted and they were significantly lower than the levels in the three non-silenced controls (Figure 8). Thus TPE is maintained in diploid cells and is not affected by change in mating-type.

### Protein Levels for These Reporters can be Controlled Either Transcriptionally or Post-transcriptionally

Flow cytometry was carried out on the strains for which RNA levels had been measured. The mode values of fluorescence were calculated and these were compared to the amounts of reporter mRNA (Table 2). These results suggest that in some situations protein levels were being determined by transcript level, while in other situations transcript level had no influence on protein level. For example, the transcript and protein levels in the *sir-*Δ strain marked at telomere XI-L were the same as in the *SIR3* strain marked at III-R (III-R is a non-silenced telomere). Thus the protein to transcript ratio for the non-silenced telomeric reporter may be constant. However, as our other results have already shown, when ploidy is changed the protein levels increase even though mRNA levels are unchanged. For the reporter at *hml* in haploid cells there seemed to be a strikingly different situation occurring when *SIR3* was deleted. In these cells there were large amounts of mRNA (or at least the approximately 0.1 kb fragment detected by PCR), but only a relatively small increase in protein compared with *SIR3* cells.

**Table 2:** Level of reporter fluorescence measured by flow cytometry and RNA level as ratio to that in haploid cells.

ploidy	*Sir3* status	Location of reporter	Fluorescence*	RNA: haploid RNA
n	wt	*hml*	1472	1
2n	wt	*hml*	2400	0.73
3n	wt	*hml*	3520	0.91
n	*sir3*Δ	*hml*	3504	654
n	wt	telomere XI L	1608	1
2n	wt	telomere XI L	2608	1.04
n	*sir3*Δ	telomere XI L	3988	5.18
n	wt	telomere III R	3536	5.41
n	wt	*URA3* native position	6608	6.18

* modal value of fluorescence. wt: wild-type.

## Discussion

We have demonstrated that transcriptional silencing at telomeres or *HML* is unaffected by ploidy or by mating-type. By measuring both RNA and protein abundance we have identified multiple levels of regulation for reporter genes located in heterochromatic regions. The presence of SIR proteins and the location of the reporter gene both affected the abundance of reporter gene RNA. In contrast, as the cells increased in ploidy, the amount of reporter gene protein increased while the levels of mRNA were unchanged, indicating post-transcriptional control. These different relationships between RNA and protein levels demonstrate that transcriptional silencing is not the only mechanism regulating gene expression in heterochromatic regions.

Our results indicate that levels of telomere and *HM* reporter proteins are elevated in diploids by a post-transcriptional mechanism. Further analysis showed that protein levels of the telomeric reporter were influenced more by *MAT* status than by ploidy. However, at *HML* there was a more subtle effect of mating-type on reporter levels. Two previous studies [20], [21] have also looked at the effects of ploidy on reporter gene expression. Mercier *et al* observed decreased 5-FOA resistance in diploid versus haploid cells when *URA3* was positioned at a telomere and concluded that silencing was decreased in diploids compared to haploids [21]. Mazor and Kupiec extended these results by showing that 5-FOA resistance of haploids could be reduced to diploid levels by expressing a1/alpha2 in the haploid cells [20]. They also showed that haploid *hmr::ADE2* cells grew better on adenine dropout plates when a1/alpha2 was expressed, suggesting that expression at *HM* loci is also influenced by *MAT* status [20]. Our results from FOA and flow cytometry assays have some agreement with the results of these studies showing that mating-type has a substantial influence on the expression of a telomeric reporter, but only minor effects on an *HM* reporter. However, our QRT-PCR analysis revealed that the affect of ploidy on reporter gene protein levels is due to a post-transcriptional effect rather than transcriptional silencing.

Mazor and Kupiec also looked at the known targets of the a1/alpha2 repressor [9] and found that deletion of both *HOG1* and *STE5* could replicate the effect of ectopic a1/alpha2 expression [20]. *HOG1* is in the osmotic stress pathway known as the high osmolarity glycerol (HOG) pathway and *STE5* is in the pheromone response pathway [22]. As a MAPK, Hog1p is known to be activated by phosphorylation and then to move into the nucleus to control the activity of several transcription factors by their phosphorylation [22]. Hog1p has also been shown to have effects on translation, including translational repression of limited groups of proteins during response to hyperosmotic shock [23], [24]. Thus it is still possible that Hog1 is involved in the post-transcriptional control of the reporters in our study.

The increase of *HM* reporter protein with ploidy was only partially determined by mating type. When fluorescence levels of the *hml::YFP* reporter were plotted against cell volume it was found that there was a simple linear relationship between the two, irrespective of whether volume had been changed by ploidy or by heterozygous deletion of dosage dependent cell size genes in the diploid cell. This suggests that there may be a general increase in protein levels relating to cell volume; a study of human cells has indicated that protein level is generally dependent on cell size over a wide size range [25]. The increase in protein expression in diploids versus haploid yeast may be required to maintain protein concentration balances in a larger cell.

Examination of chromatin structure by micrococcal nuclease digests revealed slight differences at the promoter of a telomeric reporter among strains differing by mating type or ploidy. The promoter of diploid cells resembled the open promoter structure previously described for *URA3* [6], whereas, haploids and diploids with one copy of *MAT* deleted had a more closed promoter structure. It is unclear what these promoter differences represent since transcript levels were similar for all three strains. However, there are examples of nuclear processes influencing cytoplasmic translation [26], [27], and a recent study has shown RNA polymerase II subunits, Rpb4p and Rpb7p, interact with both the mRNA during transcription and subsequently with the translation initiation factor [28]. The chromatin structure surrounding the promoter of the telomeric reporter was not altered by mating type or ploidy, suggesting that this structure is what maintains transcriptional silencing in the strains.

It is essential for haploid yeast cells to maintain silencing at the *HM* loci, otherwise these cells would express proteins of both mating-types and be unable to mate. There is not an obvious need for *HM* silencing to occur in diploids as well, so our finding that silencing is maintained is unexpected, and that the cell should also increase protein levels is even more surprising. Why the ploidy control of this protein increase is different at *HM* loci versus telomeres may also seem paradoxical. As telomeres and *HM* loci share much of the heterochromatin silencing machinery, they might be expected to have the same control of response to ploidy. They do both derepress in the absence of Sir proteins for example. One possibility is that the original control for both *HM* loci and telomeres was via mating type expression. *HM* loci are under the additional constraint of needing to be protected from cutting by the *HO* endonuclease when haploid spores undergo mating type switching. Monitoring ploidy may therefore be an important selective pressure leading to the evolution of an additional mechanism for determining ploidy at *HM* loci. Such a mechanism, independent of mating type expression would be dominant over the a/alpha effect and so we observe the retention of the diploid expression at *HM* loci in diploids with one copy of *MAT* deleted. This mechanism could directly or indirectly involve cell volume.

The results presented here reveal an example of post-transcriptional regulation in yeast and show the dangers of assuming that transcriptional silencing in yeast can always be assessed by assays that rely on protein levels. The conclusions in some of the previous literature on transcriptional silencing will now need to be re-examined, since all too often it has been assumed that reporter protein levels are a direct reflection of transcriptional levels.

## Materials and Methods

### Yeast Strains and Strain Construction

#### Details of all strains used are given in Table S1

Strains with *URA3-yEGFP* adjacent to core X of XI-L (FEP318-19) or III R (FEP318-23), or at the *URA3* native locus (PIY125) were constructed as described by Loney *et al* [6] and they were derived from strain FYBL1-8B [29]. The haploid *sir3-*Δ strain with *URA3-yEGFP* at XI-L (hERL9) was also described by Loney *et al* [6]. The diploid strain (YGL254) with a single copy of the reporter was created by mating FEP318-19 with a haploid strain YGL246 (*MATalpha ura3*-Δ). The diploid strain then had one copy of *MAT* deleted with *kanMX* to create strain YGL364. The *URA3-yEGFP* also contains 217 bp upstream of the *URA3* ORF but no sequence from downstream of the *URA3* ORF.

To create strains with *URA3* at a truncated VII-L end, haploid strains were transformed with the *SalI-EcoRI* fragment of plasmid pVII-L URA3-TEL [5]. This fragment targeted *URA3* with attached telomeric sequence to *ADH4*, resulting in truncation at this point. The resulting strains were hERL3 (S288C background) and YGL530 (Y55 background). Diploids were created by mating to strains without the telomeric reporter: YGL250 (S288C) and YGL543 (Y55). *MATa* was deleted from YGL250 to create strain YGL361 and *MATa* was inserted at *trp1-B* of YGL530 to create strain YGL532.

The strains of *S. paradoxus* were derived from a wild strain YPS138 [30]. *URA3* and *HO* were deleted, and the truncation and addition of *URA3* to VII-L was as described for other strains.

The haploid strain with *YFP* at *hml* (Y3401) and the *sir3-*Δ strain derived from this (Y3402) were created by Xu *et al* [13]. The *YFP* had a nuclear localization signal from SV40 attached to the N-terminus and was flanked by the *URA3* promoter and terminator. This construct replaces most of the *alpha2* ORF and all of the *alpha1* ORF. The diploid strain (YGL540) was created by mating Y3401 with W303-1A [31]. *MATa* or *MATalpha* were deleted from this diploid using *kanMX* to create strains YGL555 and YGL556 respectively. The triploid strain (JMM14) was derived by mating YGL555 with W303-1A. The haploid strain heterozygous for *MAT* (JMM18) was derived by integrating a *TRP1* and *MATa* containing plasmid into the *trp1-1* locus of Y3401 (see also Text S1). Single copies of *SIR3* or *SIR4* were replaced with *hphMX* to make strains JMM22 and JMM52 respectively.

To create diploid cells of different sizes the BY4741 [32] based haploid Yeast Deletion Collection [19] strains were mated to strain Y3401 and checked for non-mating phenotype. The strains used had deletions for YOR083W (*WHI5*), YAL056W (*GPB2*), YKL109W (*HAP4*), YJL127C (*SPT10*), YOR043W (*WHI2*), YDR335W (*MSN5*), YML014W (*TRM9*), or YKL114C (*APN1*),.

### 5-FOA Assays

Yeast strains were streaked for singles on complete media (COM) plates and grown for 3 days at 30°C. Single colonies from each strain were resuspended in 45 µl water and then diluted serially ten fold to 10^−5^. Dilutions were plated on COM, URA-dropout, and 5-FOA plates. Control strains were also plated; these either had *URA3* deleted, or *URA3* expressed from its wild-type location. The percentage growth on 5-FOA compared to COM, was calculated after 3 days at 30°C and taken to represent the percentage silencing in that strain.

### Flow Cytometry

Cells were grown overnight at 25°C in COM media, then diluted 1∶10 in 5 mls of COM and grown for a further 4 hours. The cells were washed twice in 1 ml PBS and resuspended in 1 ml PBS. Flow cytometry was performed on the Apogee A40 flow cytometer. Data was collected for 10000 cells per sample and analysed using Flowjo software. Data was plotted as histograms of fluorescent peak area. To plot fluorescence versus another parameter, mode values of fluorescence were used since fluorescence of populations does not show a normal distribution.

### Chromatin Analysis using Micrococcal Nuclease I

Chromatin analysis using micrococcal nuclease I (MNase I) was performed as described previously [6]. Spheroplasts were prepared from 1.2×10^9^ cells using zymolyase 100 T and permeabilized with the detergent NP-40. Chromatin from 2.0×10^8^ of these cells was digested with 2.2, 5.5 or 11 units/ml of MNase I at 37°C for 4 mins. An equivalent amount of purified DNA was digested with 5 units/ml of MNase I for 35 s at 37°C, to yield the deproteinized DNA digestion patterns. Marker DNA was obtained by digesting purified DNA from the same cells with *StuI* and *PstI* restriction enzymes. All samples were purified and analysed by indirect end-labeling [33] by digestion with *Stu*I. Digested samples were separated by agarose gel electrophoresis and transferred to nylon membranes. The MNase I digestion pattern was visualized with a 200 bp probe adjacent to the end-label digestion site. Probes were generated by radio-labeling gel-purified PCR fragments amplified from yeast genomic DNA.

### Measurement of Band Intensities from Autoradiograph of Chromatin Blot

Measurements were made using ImageJ software (http://rsbweb.nih.gov/ij/). Analysis was performed using the command “Plot profile” and the region of interest was defined with a single straight line vertically through the centre of the bands. The resulting data was plotted as intensity versus distance in inches along the line.

### Calculation of Cell Volume

Images of cells were taken using the 60× objective on a Nikon Eclipse 80i microscope. For at least 100 cells of each strain, the length of the longest axis, excluding buds, was measured using IP Lab software version 3.71 (Scanalytics). The widths were also measured and then the cell volumes were estimated by assuming that the cells were spheroid in shape.

### Quantitation of RNA by QRT-PCR

Cells were grown as for flow cytometry, but in 10 mls of COM media. Cells were counted using a haemocytometer, then RNA was extracted from equal numbers of cells using the acid phenol method [34]. Equal volumes of RNA were treated with DNaseI (Sigma) then reactions were set up with random primers (Invitrogen) and with or without reverse transcriptase (Superscript III, Invitrogen). PCR was performed using SYBR green PCR mix (Agilent Technologies) and primers for YFP (TGGAAGCGTTCAACTAGCAG and CTTTTCGTTGGGATCTTTCG) or yEGFP (TTCCATGGCCAACCTTAGTC and GCATGGCAGACTTGAAAAAG). Standard curve reactions were performed on each occasion, in triplicate using 10 fold dilutions of genomic DNA (100 ng to 0.001 ng template) from the appropriate haploid strain (FEP318-19 or Y3401). The reactions were run on the Rotor-Gene 6000 robot (Qiagen) and analysis was performed using the connected software (version 1.7, Qiagen). Negative control reactions (no reverse transcriptase) were always checked to ensure zero PCR product.

## Supporting Information

Table S1 **Yeast strains.**
(DOC)

Text S1 **Further details on strain construction.**
(DOC)
