# Supplementary material for: Apparent Ploidy Effects on Silencing Are Post-Transcriptional at HML and Telomeres in Saccharomyces cerevisiae
Source: PLoS One. 2012 Jul 9;7(7):e39044. doi: 10.1371/journal.pone.0039044 (PMC3392252; doi:10.1371/journal.pone.0039044)
Supplement: Table S1 — Yeast strains. (DOC) [file pone.0039044.s001.doc]

***Saccharomyces cerevisiae* strains**

| **Strain** | **Background** | **Genotype** | **Source** |
| --- | --- | --- | --- |
| FYBL1-8B | S288C | *MATa lys2∆202 leu2∆1 his3∆200 ura3∆851* | Fairhead *et al* 1996 |
| FEP318-19 | S288C | FYBL1-8B *URA3-yEGFP-*TELXIL | Loney *et al* 2009 |
| FEP318-23 | S288C | FYBL1-8B *URA3-yEGFP-*TELIIIR | Loney *et al* 2009 |
| PIY125 | S288C | *MATα* *lys2∆202 leu2∆1 his3∆200 ura3-52::URA3-yEGFP* | Loney *et al* 2009 |
| hERL3 | S288C | *MAT****a*** *lys2∆202 leu2∆1 ura3-52::hphMX4 adh4*::*URA3-yEGFP-*TELVIIL | Loney *et al* 2009 |
| YGL246 | S288C | *MATα* *ura3*-Δ | This study |
| YGL250 | S288C | hERL3 x YGL246 | This study |
| YGL361 | S288C | YGL250 *mata::kanMX* | This study |
| YGL254 | S288C | FEP318-19 x YGL246 | This study |
| YGL364 | S288C | YGL254 *mata*::*kanMX* | This study |
| hERL9 | S288C | FYBL1-8B *URA3-yEGFP-*TELXIL *sir3::kanMX* | Loney *et al* 2009 |
| YGL530 | Y55 | *MATalpha* *ho*Δ*pstI lys2::NdeI-2 spo13*Δ *ura3-N leu2*Δ*X-R*  *trp1-B adh4::URA3*-TELVIIL | This study |
| YGL494 | Y55 | *MATa ho*Δ*pstI ura3-N leu2*Δ*X-R ade1 can^R^  lys2-NdeI-2* | This study |
| YGL543 | Y55 | YGL530 x YGL494 | This study |
| YGL532 | Y55 | *MATalpha ho*Δ*pstI lys2::NdeI-2 spo13*Δ *ura3-N leu2*Δ*X-R*  *MATa-TRP1:*:*trp1-B adh4::URA3*-TELVIIL | This study |
| Y3401 | W303* | *MATα hml::*P*_URA3_*-*YFP hmr::*P*_URA3_-CFP* | Xu *et al* 2006 |
| Y3402 | W303* | *MATα hml::*P*_URA3_*-*YFP hmr::*P*_URA3_-CFP sir3*Δ::*LEU2* | Xu *et al* 2006 |
| W303-1A | W303 | *MATa, ade2-1, can1-100, his3-11,15, leu2-3,112, trp1-1, ura3-1* | Thomas and Rothstein 1989 |
| YGL540 | W303 | Y3401 x W303-1A | This study |
| YGL555 | W303 | YGL540 *mata*::*kanMX* | This study |
| YGL556 | W303 | YGL540 *matα*::*kanMX* | This study |
| JMM14 | W303 | YGL555 x W303-1A | This study |
| JMM18 | W303 | Y3401 *MATa-TRP1::trp1-1* | This study |
| JMM22 | W303 | YGL540 *SIR3/sir3Δ::hphMX* | This study |
| JMM52 | W303 | YGL540 *SIR4/sir4Δ::hphMX* | This study |
| BY4741 | S288C | *MATa* *his3Δ1 leu2Δ0 met15Δ0 ura3Δ0* | Brachmann *et al* |
| JMM43 |  | Y3401 x BY4741 *whi5*Δ::*kanMX* | This study |
| JMM44 |  | Y3401 x BY4741 *scp160*Δ::*kanMX* | This study |
| JMM45 |  | Y3401 x BY4741 *trm9*Δ::*kanMX* | This study |
| JMM46 |  | Y3401 x BY4741 *gpb2*Δ::*kanMX* | This study |
| JMM47 |  | Y3401 x BY4741 *spt10*Δ::*kanMX* | This study |
| JMM48 |  | Y3401 x BY4741 *msn5*Δ::*kanMX* | This study |
| JMM49 |  | Y3401 x BY4741 *apn1*Δ::*kanMX* | This study |
| JMM50 |  | Y3401 x BY4741 *whi5*Δ::*kanMX* | This study |
| JMM51 |  | Y3401 x BY4741 *hap4*Δ::*kanMX* | This study |

* Derived from W303-1B (*MATα ade2-1 his3-11,15 leu2-3,112 trp1-1 ura3-1 can1-100*) but are *ADE2.*

***Saccharomyces paradoxus* strains**

| **Strain** | **Background** | **Genotype or details** | **Source** |
| --- | --- | --- | --- |
| YPS138 |  | Associated with *Quercus* species Pennsylvania, USA. | Liti et al 2005 |
| TL192 | YPS138 | *MATα ho::hphMX ura3::kanMX adh4::URA3-*TEL VIIL | Liti et al 2009 |
| TL174 | YPS138 | *MATa/MATα ho::hphMX/ ho::hphMX ura3::kan/ura3::kan adh4::URA3-*TEL VIIL | Liti et al 2009 |
