# Supplementary material for: Apparent Ploidy Effects on Silencing Are Post-Transcriptional at HML and Telomeres in Saccharomyces cerevisiae
Source: PLoS One. 2012 Jul 9;7(7):e39044. doi: 10.1371/journal.pone.0039044 (PMC3392252; doi:10.1371/journal.pone.0039044)
Supplement: Text S1 — Further details on strain construction. (DOC) [file pone.0039044.s002.doc]

**Text S1: Strain construction**

**Construction of strain JMM18**

Plasmid pBS-TRP1-MATa was derived by inserting two sequences from other plasmids into pBluescript SK+: the *EcoRI*-*HindIII* *MATa* fragment from pRHB38 [1,2] was inserted into the *EcoRI*-*HindIII* sites, and the *EcoRI*-*BglII* *TRP1* fragment from pJH331 (J.E. Haber) was inserted adjacent to this such that the 5’ ends of *MATa* and *TRP1* were nearest to each other. pBS-TRP1-MATa was cut in *TRP1* with *Bsu36I* and integrated into *trp1-1* of Y3401. Thus in JMM18, *MATa* and *TRPI* are adjacent to *trp1-1.*

1. Borts RH, Leung WY, Kramer W, Kramer B, Williamson M, et al. (1990) Mismatch Repair-Induced Meiotic Recombination Requires the PMS1 Gene Product. Genetics 124: 573-584.

2. Borts RH, Haber JE (1989) Length and Distribution of Meiotic Gene Conversion Tracts and Crossovers in Saccharomyces cerevisiae. Genetics 123: 69-80.
